# Adaptive signal theory: an integrative evolutionary, ecological, and intergenerational framework for psychological distress

**DOI:** 10.3389/fpsyg.2026.1845443

**Published:** 2026-06-10

**Authors:** Amanda Suncire-Nerison

**Affiliations:** Department of Clinical Social Work, Private Practice, Apple Valley, MN, United States

**Keywords:** adaptive signal theory, co-regulation, generational trauma, hormesis, integrative treatment, neurobiological adaptation, neuroception, Polyvagal Theory

## Abstract

Despite substantial increases in pharmacological and psychotherapeutic treatment availability, global rates of psychological distress continue to rise, a pattern existing frameworks, taken individually, cannot fully explain. Current dominant models frequently conceptualize distress primarily as neurochemical dysfunction, emphasizing symptom reduction while leaving broader neurobiological signaling processes insufficiently addressed. For a clinically significant subset of individuals, this contributes to recurring symptom patterns, escalating treatment requirements, and eventual treatment resistance that existing literature has not yet fully accounted for. This paper introduces Adaptive Signal Theory (AST), an integrative evolutionary, ecological, and intergenerational framework proposing that psychological distress must be understood simultaneously across four nested levels: the evolutionary origin of distress signals, the autonomic mechanisms of their expression, the contemporary ecological conditions that chronically sustain them, and the intergenerational pathways through which dysregulation may transmit across generations. Critically, AST argues these levels are not parallel explanations for the same phenomenon; rather, they are nested layers of a single adaptive system, and failure to integrate them may produce predictable and clinically recognizable patterns of treatment limitation. Drawing on Polyvagal Theory (Porges, 2011), allostatic load theory (McEwen and Stellar, 1993), hormesis (Calabrese and Mattson, 2011), somatic trauma theory (Levine, 1997; van der Kolk, 2014), attachment theory (Bowlby, 1969), evolutionary psychiatry (Nesse, 2019; Hollon, 2020), and interpersonal neurobiology (Siegel, 2012), AST advances four central propositions: that psychological distress reflects functional neurobiological signaling; that exclusive symptom suppression may fail to resolve underlying threat-detection processes; that contemporary post-industrial environments may systematically deprive nervous systems of the relational, sensory, and co-regulatory inputs necessary for baseline neuroception of safety; and that chronic disruption of adaptive signal processing in caregivers may contribute to intergenerational consequences for infant neurobiological development. The perinatal period is examined as a clinically significant context in which all four propositions converge simultaneously, with implications for dyadic intervention, maternal regulatory restoration, and co-regulatory treatment approaches. A central clinical implication follows across populations and presentations: treatment effectiveness depends on alignment between the level at which a signal is generated and the level at which intervention is applied. Directions for future empirical investigation and interdisciplinary clinical application are discussed.

## Introduction

The global prevalence of psychological distress has increased significantly over recent decades, with anxiety and depressive disorders representing leading contributors to global disease burden (World Health Organization, 2017). Concurrently, the use of psychotropic medications, particularly antidepressants, has risen substantially in the United States and other developed nations (Pratt et al., 2017). Despite these advances in treatment availability, population-level outcomes have not demonstrated commensurate improvements in mental health, raising important questions regarding the adequacy of current conceptual frameworks.

The dominant psychiatric paradigm conceptualizes psychological distress primarily as dysfunction within neurochemical or neurobiological systems, often emphasizing symptom reduction through pharmacological intervention. While this model has provided meaningful relief for many individuals, it may not fully account for the persistence, recurrence, or treatment resistance observed in a significant subset of cases.

This paper proposes Adaptive Signal Theory (AST) as a theoretical synthesis framework that reexamines psychological distress through a neurobiological and evolutionary lens. Specifically, AST suggests that many forms of distress, such as anxiety, panic, depressive withdrawal, and dissociation, may represent adaptive signaling processes generated by the nervous system in response to perceived or actual threat. Within this framework, distress is not conceptualized as evidence of system failure, but rather as an indication of system activation in the service of survival. The term *adaptive intelligence* is used here to describe the nervous system’s capacity to generate context-sensitive survival responses through functional signaling processes, rather than to imply conscious or cognitive intent.

Importantly, this perspective does not deny the suffering associated with psychological distress, nor does it reject the utility of pharmacological or diagnostic models. Instead, AST seeks to expand the interpretive lens through which distress is understood, with the goal of informing more comprehensive and integrative treatment approaches. Pharmacological intervention remains a valuable component of care; however, AST proposes that such interventions may be most effective when situated within a broader framework that attends to the underlying signals driving symptom presentation.

This model integrates and extends existing theoretical frameworks, including Polyvagal Theory (Porges, 2011), somatic models of trauma (Levine, 1997; van der Kolk, 2014), interpersonal neurobiology (Siegel, 2012), and research on adaptive stress responses (Calabrese and Mattson, 2011). By synthesizing these perspectives, AST aims to provide a unifying framework for understanding psychological distress as both biologically meaningful and clinically actionable.

AST is not intended to introduce a new explanation of psychological distress in isolation, but rather to propose a structural integration of existing frameworks into a unified, multi-level clinical model with distinct implications for treatment.

## The conceptual novelty of adaptive signal theory

The question of what AST contributes beyond existing frameworks deserves a direct answer. Each of the theoretical traditions informing this paper has established foundational knowledge, and each has a specific boundary that leaves a clinically significant gap unaddressed.

Polyvagal Theory (Porges, 2011) explains how the autonomic nervous system detects and responds to threat. It does not fully explain why, decades after that detection, a patient receiving appropriate pharmacological and cognitive treatment continues to deteriorate. Internal Family Systems (Schwartz, 1995) positions symptoms as protective parts with positive intent but operates at the intrapsychic level and does not fully account for the neurobiological or ecological processes of how those protective responses are generated or sustained. Evolutionary psychiatry, as articulated by Nesse (2019) and Hollon (2020), explains why depression and anxiety exist as evolved features rather than design flaws. It does not provide a clinical model for what to do when those features are chronically activated in a post-industrial environment that bears no resemblance to the conditions under which they evolved. Somatic trauma theory (Levine, 1997; van der Kolk, 2014) explains how traumatic experience is encoded subcortically and why cortically targeted interventions do not reach it. It does not extend to explaining the intergenerational dimension, why a caregiver’s unresolved activation directly compromises the neurobiological development of a child who has experienced no trauma of their own. Interpersonal neurobiology (Siegel, 2012) explains how co-regulatory disruption in early development produces lasting deficits in self-regulation. It does not clearly connect that developmental mechanism to the clinical phenomenon of treatment resistance in adults, or to the ecological conditions that systematically deprive contemporary nervous systems of the co-regulatory inputs required for baseline safety. And while Schroder et al. (2023) have now provided preliminary empirical support for the value of functional reframing as a clinical intervention, their work addresses one technique; it does not offer an integrative theoretical framework that would explain when that technique is indicated, why it works, what must accompany it, and how the clinician’s own regulatory state determines its effectiveness.

These frameworks converge on a shared limitation, approached from a different direction: no existing model fully integrates the evolutionary origin of distress, the autonomic mechanism through which it is expressed, the environmental conditions that chronically sustain it, the developmental pathway through which it transmits across generations, and the clinical implications of all four, in a single framework with coherent propositions about treatment. A clinician working with a treatment-resistant, socially isolated postpartum patient with a complex trauma history can draw on Porges to understand her autonomic state, on Levine to understand why talk therapy has not helped, on Siegel to understand her infant’s risk, and on Nesse to understand why her distress is not a malfunction. But none of those frameworks, individually or listed side by side, tells the clinician how these mechanisms interact, which intervention should come first, why the therapeutic relationship is not merely supportive but neurobiologically primary, or how the same ecological forces driving her isolation may also be sustaining her nervous system’s inability to register safety regardless of what the therapy room provides.

Adaptive signal theory is a theoretical synthesis framework. Its contribution is not a new empirical discovery but a new structural argument: that the evolutionary, autonomic, ecological, and intergenerational dimensions of psychological distress are not parallel explanations for the same phenomenon, they are nested layers of a single adaptive system, and understanding them as such changes both what questions the clinician asks and what interventions logically follow. The four pillars of AST are the clinical propositions that emerge specifically from treating these levels as integrated rather than separate. Each pillar is a hypothesis that no single existing framework generates, because each requires the intersection of at least two levels of analysis that the prior literature has not yet combined into a unified clinical model.

The contribution of Adaptive Signal Theory is not the proposal that psychological distress is adaptive, a position well established across multiple existing frameworks, but the articulation of how this principle translates into a unified, multi-level clinical model.

Specifically, AST advances the argument that psychological distress must be understood simultaneously across evolutionary, autonomic, ecological, and intergenerational levels, and that failure to integrate these levels results in predictable patterns of treatment limitation. From this integration emerges a central clinical implication: treatment effectiveness depends on alignment between the level at which a signal is generated and the level at which intervention is applied.

In this sense, AST does not introduce a new explanation of distress in isolation, but rather a structural framework that organizes existing explanations into a coherent model with direct implications for clinical decision-making (Table 1).

**Table 1:** Adaptive signal theory in relation to existing frameworks: explanatory scope and contribution.

Framework	What it explains	Where it stops	AST contribution at this level
Polyvagal Theory (Porges, 2011)	How the autonomic nervous system detects and responds to safety and threat through hierarchically organized circuits; the mechanism of neuroception below conscious awareness	Does not explain why decades of appropriate treatment fail to resolve activation, nor why the ecological environment sustains threat neuroception regardless of therapeutic intervention	Pillar 1: Provides the autonomic mechanism. AST extends Polyvagal Theory into ecological and intergenerational levels that the original framework does not address.
Evolutionary Psychiatry (Nesse, 2019; Hollon, 2020)	Why depression, anxiety, and related states exist as evolved features rather than design flaws; the adaptive function of negative affect in ancestral environments	Does not provide a clinical model for what to do when evolved features are chronically activated in post-industrial environments bearing no resemblance to ancestral conditions	Pillars 1 and 3: Provides the evolutionary rationale. AST adds the ecological mismatch model and the clinical translation that evolutionary psychiatry leaves undeveloped
Somatic Trauma Theory (Levine, 1997; van der Kolk, 2014)	How traumatic experience is encoded subcortically as incomplete biological responses; why cortically targeted interventions do not reach it; the role of somatic completion in resolution	Does not extend to the intergenerational dimension: why a caregiver’s unresolved activation directly compromises the neurobiological development of a child with no trauma of their own	Pillar 2: Provides the subcortical mechanism. AST names the biological debt dynamic and extends the incomplete-cycle logic across generations
Interpersonal Neurobiology (Siegel, 2012)	How regulatory capacity is co-constructed through early relational experience; the developmental consequences of disrupted co-regulation; the neurobiological basis of attachment	Does not connect developmental co-regulatory disruption to treatment resistance in adults, or to the ecological conditions that systematically deprive contemporary nervous systems of co-regulatory inputs	Pillar 4: Provides the developmental mechanism. AST connects it to treatment resistance and ecological conditions in adults, and to the intergenerational propagation pathway
Functional Reframing (Schroder et al., 2023)	Preliminary RCT evidence that reframing depression as functional signal rather than disease improves patient outcomes; empirical support for the core reframing principle	Addresses one technique in isolation; does not offer a multi-level theoretical framework explaining when reframing is indicated, why it works, what must accompany it, or how the clinician’s regulatory state determines its effectiveness	All four pillars: Provides the theoretical architecture that explains why Schroder’s reframing works and situates it within a comprehensive clinical model
Adaptive signal theory (AST)	Contribution: The first framework to integrate the evolutionary, autonomic, ecological, and intergenerational dimensions of psychological distress as nested layers of a single adaptive system, with coherent clinical propositions derived specifically from that integration. AST does not replace any of the frameworks above; it provides the structural architecture within which their contributions are organized, sequenced, and extended. Constitutional presentations, including bipolar spectrum and schizophrenia spectrum conditions, fall outside AST’s defined scope.		

AST = Adaptive Signal Theory; RCT = randomized controlled trial. Frameworks listed represent the primary theoretical traditions informing AST. The limitation column identifies the specific explanatory boundary at which each framework stops and AST begins, rather than a general critique of the framework’s validity or scope within its own domain.

## Theoretical background and existing frameworks

### Polyvagal Theory

Stephen Porges’s Polyvagal Theory (Porges, 2011) established a hierarchical model of autonomic nervous system function that substantially reframed the understanding of physiological stress responses. Rather than a simple binary fight-or-flight model, Porges proposed three phylogenetically ordered circuits: the ventral vagal complex, associated with social engagement and safety; the sympathetic nervous system, associated with mobilization; and the dorsal vagal complex, associated with immobilization or conservation of resources.

Central to Polyvagal Theory is the concept of neuroception, the nervous system’s capacity to detect cues of safety and threat below the threshold of conscious awareness (Porges, 2011). Neuroception operates continuously, scanning both internal and external environments for signals that inform autonomic state. Critically, neuroception does not respond to what a person cognitively believes; it responds to what the nervous system detects. This distinction carries significant clinical implications for treatment models that rely primarily on cognitive or pharmacological intervention without attending to the underlying neuroceptive processes driving activation.

Polyvagal Theory established that the autonomic nervous system functions as a detection and response system, organized around the fundamental imperative of survival. AST extends this foundation by proposing that when the nervous system generates states of anxiety, panic, or hypervigilance, it is completing the function it was designed to complete: signaling that something in the organism’s environment or internal landscape may warrant attention, processing, or a change in conditions.

### Allostatic load and the physiology of stress responses

McEwen and Stellar’s allostatic load model (McEwen and Stellar, 1993) provided a foundational account of how the body’s stress response architecture functions as an organized, adaptive system rather than a source of random dysregulation. Allostasis, the process by which the organism maintains stability through change, involves coordinated activation across cardiovascular, neuroendocrine, and immune systems. Physiological responses including autonomic hyperarousal and elevated cortisol reactivity reflect calibrated biological preparation rather than system failure. McEwen’s subsequent research demonstrated that when the organism is chronically activated but the adaptive load is never discharged, the regulatory setpoint shifts: the baseline threat threshold rises, and the system requires escalating activation to produce the same defensive response. This cumulative biological cost of sustained activation, termed allostatic load, is measurable and has well-documented downstream effects on neurological, cardiovascular, and immune functioning (McEwen, 1998).

This framework carries direct implications for the clinical population AST addresses. Physiological responses that may appear dysfunctional, including hypervigilance, exaggerated startle responses, and chronic autonomic arousal, may represent the activation of precision survival architecture in response to relational, developmental, and existential threats. Allostatic load theory further provides the mechanistic basis for AST’s Pillar Two: when suppression of adaptive signals prevents the discharge of accumulated activation, the regulatory setpoint continues to shift, potentially contributing to the escalating symptom burden observed in treatment-resistant presentations. This reframing, from dysfunction to calibrated and cumulative survival response, is the empirical foundation on which Pillars One and Two of AST rest.

### Hormesis and biological stress adaptation

Hormesis refers to the well-documented biological phenomenon whereby organisms demonstrate adaptive responses to low to moderate doses of stressors that would be harmful at higher doses (Calabrese and Mattson, 2011). The principle has been demonstrated across multiple biological systems: bones demonstrate increased density under mechanical load, immune systems develop through calibrated pathogen exposure, neurons form new synaptic connections through cognitive challenge, and cardiovascular systems develop efficiency through aerobic stress. The removal of all challenge does not produce optimal biological functioning; it may produce attenuated capacity.

Research on posttraumatic growth further supports this principle in the psychological domain. Tedeschi and Calhoun (2004) documented that a substantial proportion of trauma survivors report increased personal strength, deepened relational connections, enhanced appreciation for life, and spiritual development following survival of significant adversity. This body of research suggests that the relationship between hardship and psychological functioning is not simply linear, that exposure to manageable adversity may, under appropriate conditions, facilitate development rather than preclude it. In this respect, posttraumatic growth functions as a psychological analogue to hormesis: adaptive capacity emerging through, rather than despite, the encounter with stress, provided the stress does not exceed the organism’s regulatory threshold.

### Somatic trauma theory and interpersonal neurobiology

Levine’s somatic experiencing model (Levine, 1997) proposed that trauma may be understood not primarily as an event but as an incomplete biological response, a survival response that was mobilized but not fully discharged. The therapeutic implications of this model suggest that healing may require not suppression of physiological activation but its completion through titrated somatic processing. Levine’s work provides a mechanistic bridge between the signal hypothesis of AST and clinical intervention.

Van der Kolk’s synthesis of trauma neuroscience (van der Kolk, 2014) demonstrated that traumatic experience is encoded somatically, in the body and in subcortical brain structures, rather than primarily in cortical narrative systems. This has significant implications for treatment models that target cortical neurochemistry: such approaches may address the conscious experience of distress while leaving subcortical signal architecture intact and continuing to generate activation. An integrative approach that addresses both cortical and subcortical levels of processing offers more comprehensive outcomes.

Siegel’s interpersonal neurobiology (Siegel, 2012) established that the nervous system develops its regulatory capacity primarily through co-regulation with attuned caregivers. The capacity for self-regulation is not fully innate; it is significantly co-constructed through relational experience. This framework provides the theoretical foundation for AST’s fourth pillar regarding the potential intergenerational implications of disrupted co-regulatory relationships.

## Adaptive signal theory: core propositions

AST is organized around four interrelated propositions that together constitute a framework for recontextualizing psychological distress from primary pathology to adaptive biological intelligence. Each proposition is advanced as a theoretical hypothesis subject to empirical investigation rather than as established clinical fact.

### Pillar one: psychological distress reflects functional signaling processes

The first and foundational proposition of AST is that many symptom states currently classified as anxiety disorders, panic disorder, major depressive disorder, and stress-related conditions may, in significant part, reflect adaptive biological signals rather than representing primary pathologies. This proposition does not apply universally to all psychiatric presentations, particularly those with strong constitutional or genetic components, and is not intended to minimize the genuine suffering associated with these conditions.

Within the polyvagal framework, each major symptom cluster can be mapped to a specific survival function. Panic and acute anxiety may reflect sympathetic mobilization signals: the organism has detected threat and is preparing for defensive action. Generalized anxiety and hypervigilance may reflect chronic neuroception of environmental unsafety, wherein the nervous system maintains a sustained scanning state consistent with historical experience of unpredictable threat. Depression and emotional numbing may reflect dorsal vagal conservation responses: the organism has assessed that mobilization is not feasible and has reduced metabolic expenditure accordingly. Dissociation may reflect extreme protective disconnection in response to overwhelming threat.

This mapping does not minimize the suffering inherent in these states, nor does it suggest that diagnosis or treatment is unwarranted. Rather, it recontextualizes the signal as potentially meaningful, as carrying information about the organism’s current or historical threat environment that may be clinically relevant to treatment planning. The implication is that treatment approaches which attend to the signal content, alongside symptom management, may offer more comprehensive outcomes for certain presentations.

### Pillar two: exclusive reliance on symptom suppression may not resolve underlying processes

The second proposition of AST addresses the potential limitations of treatment approaches that focus primarily on symptom reduction without attending to underlying neuroceptive processes. This proposition is framed as a hypothesis regarding a subset of treatment presentations, specifically, those characterized by persistent, recurrent, or treatment-resistant symptom activation, rather than as a universal critique of pharmacological intervention.

Porges (2011) established that neuroception operates on principles of safety detection below conscious awareness. When a threat, whether external, relational, or existential, is detected, the nervous system generates activation accordingly. This activation is not extinguished by reduction of symptom experience alone; it is resolved through the organism’s neuroceptive assessment that conditions of safety have been restored. The critical distinction is between experiential symptom reduction and genuine neuroception of safety.

Adaptive signal theory proposes that for a subset of presentations, pharmacological interventions that reduce the experiential intensity of distress may not, in isolation, produce the neuroception of safety necessary to resolve the underlying activation. If the neuroceptive assessment of threat remains unaddressed, the underlying signal architecture may continue to generate activation, potentially contributing to the pattern of symptom recurrence and escalating treatment requirements observed in some individuals with treatment-resistant presentations. AST names this dynamic the biological debt mechanism: each cycle of signal suppression without signal resolution leaves an increment of unresolved activation that the nervous system must continue to manage. Over repeated cycles, the organism’s regulatory baseline shifts, consistent with McEwen’s allostatic load model (McEwen and Stellar, 1993; McEwen, 1998), such that progressively greater suppressive effort is required to maintain functional stability. The result is a predictable pattern of escalating symptom intensity, increasing medication requirements, and eventual treatment resistance that the biological debt mechanism, rather than patient non-compliance or biological refractoriness, may help explain.

This proposition has potential clinical implications for the conceptualization of treatment resistance. Current models frequently locate treatment resistance within the patient, as biological non-response or inadequate medication adherence. AST suggests an alternative hypothesis: that some treatment-resistant presentations may reflect a mismatch between the intervention modality and the neurobiological level at which the signal is being generated. Subcortically generated threat signals may require subcortically targeted interventions, such as somatic processing, EMDR, or body-based trauma approaches, to achieve resolution.

### Pillar three: contemporary environmental conditions may contribute to chronic threat activation

The third proposition of AST addresses environmental conditions that may contribute to chronic nervous system activation currently being treated as psychiatric pathology. This proposition is ecological in scope, examining the fit between the environments in which human nervous systems evolved and the environments in which they currently operate.

Porges (2011) established that neuroception scans for safety across three domains: the external environment, internal physiological states, and relational cues from other organisms. The human nervous system evolved within conditions that provided specific regulatory inputs across all three domains: relatively predictable natural rhythms, tribal social interdependence and co-regulation, embodied engagement with physical environments, and sustained access to attuned relational connection.

Contemporary post-industrial environments systematically alter many of these regulatory inputs. Chronic social isolation and relational atomization reduce access to co-regulatory resources. Economic unpredictability and continuous information exposure may compromise the establishment of safety baselines. Sedentary and predominantly indoor environments remove the body from physical and sensory inputs associated with safety and resource adequacy. Performance-oriented social environments may generate sustained social threat detection, ongoing assessment of belonging, adequacy, and status, that the nervous system processes as existential threat.

Research supports associations between these environmental factors and psychological distress. Cacioppo and Hawkley (2010) documented that chronic loneliness produces physiological changes, including elevated cortisol reactivity, disrupted sleep architecture, and increased inflammatory markers, consistent with chronic threat activation. Twenge et al. (2018) documented significant increases in depression, anxiety, and loneliness among adolescents associated with increased digital media use and decreased face-to-face social contact. Cross-cultural psychiatric epidemiology provides perhaps the most compelling evidence for the mismatch hypothesis: the World Health Organization’s international studies on schizophrenia outcomes documented significantly better functional recovery in non-industrialized nations than in developed countries, a finding that has proven robust across multiple replications (Leff et al., 1992; Hopper et al., 2007). If these conditions were purely neurobiological in origin, such differences would be difficult to explain. The ecological mismatch framing offers a coherent account: recovery appears to be supported by the relational density, structured social roles, and reduced isolation that non-industrial environments more reliably provide.

To strengthen the clinical and theoretical precision of this proposition, AST identifies three specific dimensions of environmental mismatch that are particularly relevant to contemporary presentations. First, threat duration mismatch: the nervous system evolved to respond to acute, time-limited threats that resolved through physical action; contemporary stressors, including financial precarity, occupational demands, and relational conflict, are chronic and rarely resolve through the mobilization responses the system generates. Second, social context mismatch: the nervous system evolved within conditions of tribal interdependence and sustained co-regulatory contact; post-industrial atomization systematically reduces access to the relational inputs required for baseline neuroception of safety (Bowlby, 1969). Third, completion mismatch: ancestral threats were often resolved through physical engagement that discharged accumulated activation; modern threat encounters are managed through cognitive containment and behavioral suppression, leaving activation incomplete. Each dimension of mismatch generates a distinct pattern of chronic activation, and each implies a distinct level of clinical intervention beyond symptom management.

AST proposes that these findings may reflect, in part, the accurate detection by the nervous system of environments that are deficient in inputs required for neuroception of safety. Under this framework, a clinically comprehensive response to psychological distress would attend not only to symptom management but also to the environmental, relational, and social conditions that may be sustaining nervous system activation. This ecological proposition extends the hormesis principle introduced earlier: just as organisms require calibrated stress inputs to develop adaptive capacity (Calabrese and Mattson, 2011), they require calibrated safety inputs, co-regulation, relational connection, embodied engagement, to maintain it. Contemporary environments may be systematically depleting the second without providing adequate compensation.

### Pillar four: disruptions in co-regulatory processes may have intergenerational implications

The fourth proposition of AST addresses the potential developmental and intergenerational implications of chronic disruption to adaptive signal processing. This proposition draws heavily on Siegel’s interpersonal neurobiology (2012) and the emerging literature on intergenerational trauma transmission.

Siegel (2012) established that the developing nervous system acquires its self-regulatory capacity through co-regulation, the repeated experience of having arousal states met, matched, and modulated by an attuned caregiver. This process is not supplemental to neurological development; it is a primary mechanism through which prefrontal regulatory architecture develops its capacity to modulate subcortical arousal. The quality of co-regulatory experience in early development has demonstrated associations with emotional regulation capacity across the lifespan.

Adaptive signal theory proposes that when a primary caregiver’s adaptive signal processing is chronically disrupted, whether through unresolved trauma, environmental stressors, or treatment approaches that primarily suppress symptom expression without facilitating genuine regulatory restoration, the quality of co-regulatory availability may be affected. A caregiver who is neurobiologically unable to access and process their own activation states may experience limitations in providing authentic co-regulatory attunement to a developing child, even in the absence of intentional neglect or harm.

This proposition intersects with the growing literature on intergenerational trauma transmission. Yehuda et al. (2016) documented evidence of epigenetic transmission of stress reactivity patterns in descendants of Holocaust survivors, suggesting that trauma’s neurobiological impact may involve mechanisms extending beyond behavioral modeling. AST proposes that the disruption of adaptive signal processing across generations, without resolution of the underlying threat assessments driving activation, may be a clinically underrecognized mechanism in the perpetuation of transgenerational dysregulation patterns (Table 2).

**Table 2:** Adaptive signal theory: clinical framework organized by pillar, presentation, neurobiological explanation, and indicated treatment approaches.

Pillar	Symptom / issue	AST explanation	Treatment approach
Pillar 1: signal, not disease	Acute anxiety; panic; hypervigilance; depressive withdrawal; dissociation; shame about symptoms	Symptom states reflect functional activation of evolutionarily conserved survival circuits, including sympathetic mobilization, dorsal vagal conservation, and protective disconnection, in response to detected threat. The signal carries information; the system is not broken but demonstrating adaptive intelligence.	Signal-decoding clinical inquiry (What is this nervous system detecting?); psychoeducation reframing distress as adaptive intelligence; somatic experiencing; body-based awareness; signal-informed treatment planning
Pillar 2: biological debt	Treatment resistance; symptom escalation; medication tolerance; recurrent episodes; escalating medication requirements	Pharmacological suppression reduces signal amplitude without producing neuroception of safety. Unresolved subcortical threat detection continues generating activation, creating a compounding biological debt, reflected in escalating signal intensity despite increasing intervention. The regulatory setpoint shifts over repeated suppression cycles (McEwen and Stellar, 1993), requiring progressively greater effort to maintain functional stability.	EMDR targeting subcortical threat encoding; sensorimotor psychotherapy; completion of interrupted survival responses; somatic experiencing; pharmacological intervention repositioned as therapeutic window enabling signal-processing work, not standalone resolution
Pillar 3: civilizational mismatch	Chronic anxiety; social isolation; burnout; diffuse low-grade dysregulation; generalized threat activation without identifiable acute trauma history	Contemporary environments are structurally deficient in the relational, physical, and sensory inputs required for neuroception of safety. The nervous system accurately detects social atomization, chronic unpredictability, and performance threat, producing sustained activation that reflects a pathogenic environment, not a broken organism.	Environmental and relational remediation as clinical priority; community and social connection interventions; nature exposure; embodied movement; rhythm restoration; reduction of digital and performance-oriented environmental inputs; psychoeducation about ecological contributors to dysregulation
Pillar 4: Intergenerational Dysregulation	Early developmental dysregulation; attachment disruption; transgenerational trauma patterns; parenting difficulties; parent-infant attunement failure	Chronic suppression of caregiver adaptive signals, whether pharmacologically or behaviorally, limits authentic co-regulatory availability. The developing child’s nervous system, deprived of attuned co-regulation, builds reduced self-regulatory architecture. Epigenetic transmission may further propagate stress reactivity patterns across generations (Yehuda et al., 2016).	Caregiver-focused regulatory restoration (not symptom suppression alone); dyadic and parent-infant therapy (e.g., Circle of Security, Watch Wait Wonder); intergenerational trauma processing; attachment-informed parenting support; perinatal intervention (see Table 3)

AST = Adaptive Signal Theory. Treatment approaches are indicated as clinically relevant directions rather than prescriptive protocols. Pharmacological intervention is not contraindicated within the AST framework; it is repositioned as most effective when functioning as a therapeutic window enabling signal-processing work rather than as a standalone resolution. EMDR = Eye Movement Desensitization and Reprocessing.

## Perinatal application: a clinically significant context

The postpartum period represents a particularly relevant context for the application of Adaptive Signal Theory. The transition to parenthood involves simultaneous activation of multiple neurobiological systems: significant hormonal reorganization, sleep architecture disruption, activation of attachment systems, and the existential confrontation with responsibility for a vulnerable dependent. For individuals with histories of developmental trauma, insecure attachment, or chronic threat exposure, these activations may occur against a background of nervous systems already calibrated toward heightened threat detection.

Within the AST framework, postpartum anxiety and depression may be understood, at least in part, as the activation of threat-detection systems in response to genuinely high-stakes circumstances, rather than solely as symptoms of neurochemical dysregulation. The fear of loss, the reactivation of attachment wounds, and the social isolation frequently accompanying new parenthood all represent legitimate inputs to nervous systems organized around survival and protection of what matters most.

This recontextualization carries significant implications for perinatal treatment. An integrative approach, one that addresses both symptom management and the underlying signal content driving activation, may offer more comprehensive outcomes than pharmacological intervention alone. Treatment modalities that support nervous system co-regulation, attachment security, and the explicit processing of threat-related activation may be particularly indicated for postpartum presentations in individuals with complex trauma histories.

Additionally, the co-regulatory implications of Pillar Four are particularly salient in the perinatal context. The quality of the caregiver’s nervous system regulation in the postpartum period has direct relevance to the developing infant’s neurobiological experience. Treatment approaches that support genuine restoration of regulatory capacity, rather than symptom suppression alone, may carry benefits that extend beyond the individual parent to the developing child (Table 3).

**Table 3:** Adaptive signal theory applied to perinatal mental health: assessment-to-intervention framework across four clinical phases.

Phase	Symptom / Presentation	AST explanation	Treatment approach
Phase 1: signal assessment	Postpartum anxiety; hypervigilance; depressive withdrawal; attachment fears; intrusive thoughts	Postpartum distress reflects adaptive threat-detection in high-stakes circumstances, not maternal failure. Clinical inquiry begins with the signal: What is this nervous system detecting? Assessment covers developmental trauma, attachment history, prior perinatal loss, and environmental stressors including social isolation and economic unpredictability.	AST-informed psychoeducation reframing activation as meaningful signal; individual therapy; perinatal group; prenatal consultation where possible
Phase 2: safety establishment	Chronic dysregulation; inability to access calm; somatic hyperarousal; difficulty being present with infant	Therapeutic relationship functions as the primary safety signal; attuned presence precedes technique. Co-regulation with the infant requires the caregiver’s own regulatory access first. Sensory and physical environmental inputs continuously shape neuroception of safety.	Somatic grounding; titrated resource anchoring; sensory regulation (aromatherapy, weighted items, rhythmic movement); safe-space designation for maternal downregulation; modification of nursery and home environment; individual therapy and home practice
Phase 3: signal processing	Treatment-resistant postpartum depression or anxiety; recurrent perinatal grief; reactivated attachment wounds; identity disruption of new parenthood	Prior loss and attachment wounds represent incomplete signal processing reactivated by the perinatal context. Resolution requires processing, not suppression. Pharmacological intervention, when indicated, is most effective as a therapeutic window enabling processing rather than as standalone signal suppression.	EMDR or somatic EMDR for reactivated attachment wounds; somatic experiencing for interrupted survival responses; grief processing for perinatal loss and identity transitions; attachment-focused exploration of prior history; individual therapy separate from infant-present sessions
Phase 4: dyadic co-regulation	Parent-infant attunement difficulties; infant regulatory disturbance; attachment disruption; transgenerational transmission risk	The infant communicates via autonomic and behavioral signal. Teaching signal-reading reduces anxious misinterpretation and supports attunement. Parent practices self-regulation visibly, providing the infant with repeated experience of activated-then-restored states. Addressing caregiver attachment history prevents intergenerational perpetuation.	Infant autonomic state vocabulary; Circle of Security; Watch Wait Wonder; dyadic interaction observation and feedback; regulatory modeling; intergenerational trauma processing; parent-infant dyadic sessions; attachment-informed parenting groups
Target outcomes	Individual: Maternal regulatory restoration, defined as genuine neuroception of safety enabling authentic co-regulatory availability to the infant. Dyadic: Infant nervous system receives attuned co-regulation, building prefrontal regulatory architecture associated with lifelong emotional resilience; secure attachment established. Intergenerational: Resolved maternal threat neuroception terminates transmission of stress reactivity patterns across generations.		

This table depicts a clinical conceptual framework, not a rigid sequential protocol. Phases may occur concurrently and non-linearly depending on presentation severity, trauma history, and available support systems. Pharmacological intervention is not contraindicated and may be indicated at any phase; within the AST framework it is most effectively positioned as facilitating genuine regulatory restoration rather than symptom suppression alone. All processing-oriented work (Phase 3) should be preceded by adequate regulatory stabilization (Phase 2). EMDR = Eye Movement Desensitization and Reprocessing. AST = Adaptive Signal Theory.

## Clinical implications

Adaptive signal theory does not advocate for the elimination of pharmacological treatment in mental health care. Pharmacological intervention represents a meaningful and often necessary component of comprehensive care for individuals experiencing significant psychological distress. Rather, AST proposes a reorientation of treatment philosophy that situates pharmacological intervention within a broader framework attending to underlying neurobiological signal processes.

### From symptom suppression to signal-informed practice

The primary clinical implication of AST is a proposed expansion of the initial clinical inquiry. In addition to the question, what symptoms need to be addressed? AST invites clinicians to consider: what may this nervous system be detecting, and what conditions might facilitate genuine neuroception of safety? This expanded inquiry does not replace symptom-focused assessment but may enrich treatment planning, particularly for presentations characterized by persistence or treatment resistance.

Within this framework, pharmacological intervention, when indicated, is positioned as potentially most effective when it serves to create a therapeutic window, reducing signal intensity sufficiently to allow for the engagement of processing-oriented treatment modalities, rather than as a standalone resolution of underlying activation. This integrative positioning is consistent with emerging research on combined pharmacological and psychotherapeutic treatment approaches (Cuijpers et al., 2019).

### The therapeutic relationship as co-regulatory context

Adaptive signal theory places particular emphasis on the therapeutic relationship as a primary context for co-regulatory experience. For individuals whose symptom presentation is rooted in developmental disruption of regulatory capacity, the consistent experience of having their nervous system states met with attuned presence, rather than with urgency to suppress or correct, may itself constitute a primary therapeutic mechanism.

This framework supports clinical approaches that prioritize relational safety as a prerequisite for technique-based intervention, that reconceptualize client emotional activation within sessions as clinically meaningful information rather than as treatment interference, and that attend to the therapist’s own regulatory state as a variable in the co-regulatory field.

### Treatment considerations for complex and treatment-resistant presentations

Adaptive signal theory offers a potential reframe for clinicians working with individuals whose presentations have not responded adequately to standard pharmacological or cognitive-behavioral approaches. Rather than attributing non-response to patient characteristics alone, the AST framework invites examination of whether treatment has adequately addressed the subcortical signal architecture driving symptom presentation. Referral to or integration of body-based trauma modalities, including somatic experiencing, sensorimotor psychotherapy, or EMDR, may be indicated for presentations in which cortically targeted interventions have demonstrated limited efficacy. Importantly, this recommendation does not preclude concurrent pharmacological intervention; for many individuals, medication that reduces signal intensity sufficiently to permit engagement with somatic processing represents the most effective integrative sequence.

## Limitations and future directions

Adaptive Signal Theory is a theoretical framework in early development, and several significant limitations must be transparently acknowledged.

First, the four propositions of AST are advanced as theoretical hypotheses rather than empirically established claims. The framework draws on established research in related domains (e.g., Polyvagal Theory, somatic trauma theory, hormesis, interpersonal neurobiology) but has not itself been subjected to direct empirical investigation. Rigorous empirical testing of each proposition is a prerequisite for clinical application beyond the level of theoretical orientation.

Second, AST is designed to explain environmentally and relationally generated signal dysregulation, specifically presentations in which psychological distress has been shaped primarily by developmental experience, relational disruption, or ecological mismatch. Constitutional presentations, including bipolar spectrum conditions, schizophrenia spectrum presentations, and severe obsessive-compulsive disorder, represent a different signal architecture: one that is genetic and neurobiological in origin rather than primarily experiential. These presentations fall outside AST’s defined scope, not because the framework breaks down, but because they require a different explanatory model. This boundary condition sharpens rather than weakens AST: a framework that attempts to explain all psychiatric presentations explains none of them precisely. Practitioners should exercise caution in applying AST perspectives to constitutionally determined conditions, and future work should clearly operationalize criteria for distinguishing experiential from constitutional signal dysregulation in clinical assessment.

Third, the proposition that exclusive reliance on symptom suppression may fail to resolve underlying processes, while theoretically consistent with polyvagal principles, requires direct empirical investigation. Clinical observation supports patterns consistent with this hypothesis, but controlled longitudinal research is required before clinical recommendations can be drawn.

Fourth, AST’s ecological analysis of contemporary environments as contributing to chronic threat activation is supported by correlational evidence but is subject to concerns regarding reverse causation and confounding variables. Longitudinal and cross-cultural research designs are required to more rigorously examine these relationships.

Priority areas for future empirical research include: longitudinal studies examining treatment outcomes for individuals receiving pharmacological intervention with and without concurrent signal-processing therapeutic modalities; neuroimaging studies examining subcortical versus cortical locus of activation in treatment-resistant presentations; developmental studies examining associations between caregiver regulatory restoration and infant co-regulatory development; cross-cultural studies examining relationships between community social structure and rates of anxiety and depressive presentations; and the development of clinician-facing assessment tools for identifying presentations that may be most responsive to signal-informed treatment approaches.

## Conclusion

The persistence and growth of psychological distress at a population level, despite substantial increases in treatment availability, represents a significant challenge to the mental health field and warrants ongoing theoretical examination. Adaptive Signal Theory offers a theoretical synthesis framework that recontextualizes many forms of psychological distress as adaptive signaling processes that reflect the nervous system’s adaptive intelligence: its capacity to generate meaningful activation in response to real, perceived, or historically encoded threat, rather than evidence of system failure. The signaling processes through which this intelligence operates are biological, ancient, and precise.

This framework does not propose the abandonment of current diagnostic or pharmacological models, which have demonstrated meaningful efficacy for many individuals. It proposes their expansion, an integrative orientation that attends to both the symptom and the signal, to both the experience of distress and the neurobiological processes generating it.

The clinical implications of this reorientation are significant. Treatment approaches that support genuine neuroception of safety, through relational co-regulation, somatic processing, environmental and relational restoration, and the explicit decoding of threat-activation signals, may offer outcomes that extend beyond symptom management to the restoration of the nervous system’s inherent capacity for regulation and resilience.

The human nervous system is an evolutionarily ancient, extraordinarily sophisticated biological system organized around the fundamental imperative of survival. Its signals, however uncomfortable, carry information. Adaptive Signal Theory proposes that attending to that information, recognizing it as biologically meaningful rather than pathological malfunction, rather than suppressing it, may represent the most clinically comprehensive path toward genuine and lasting restoration of wellbeing. If the rising global prevalence of psychological distress reflects, even in part, the accurate detection by millions of nervous systems of environments that are genuinely deficient in the conditions required for safety, then the most consequential clinical and public health response is not a better suppression strategy. It is a framework that takes the signal seriously. A direct clinical implication follows: for a clinically significant subset of individuals with persistent or treatment-resistant presentations, current treatment paradigms may be managing the downstream consequences of unresolved activation rather than addressing the activation itself. This is not an argument against pharmacological intervention; it is an argument for situating it within a broader model that attends to the signal the symptom is carrying. AST is offered as a step toward that framework.

## Author’s note

Amanda Suncire-Nerison, MSW, LICSW, is a licensed independent clinical social worker and trauma specialist in private practice. Her clinical work focuses on trauma, anxiety, perinatal mental health, attachment, and mood disorders, with an emphasis on integrative and neurobiologically informed approaches to treatment. She has advanced training in multiple evidence-based therapeutic modalities related to trauma processing, nervous system regulation, and relational healing. The author reports no conflicts of interest.
